# Reformulating Bread Using Sprouted Pseudo-cereal Grains to Enhance Its Nutritional Value and Sensorial Attributes

**DOI:** 10.3390/foods11111541

**Published:** 2022-05-24

**Authors:** Luz María Paucar-Menacho, Wilson Daniel Simpalo-López, Williams Esteward Castillo-Martínez, Lourdes Jossefyne Esquivel-Paredes, Cristina Martínez-Villaluenga

**Affiliations:** 1 Departamento de Agroindustria y Agronomía, Facultad de Ingeniería, Universidad Nacional del Santa, Chimbote 02711, Peru; luzpaucar@uns.edu.pe (L.M.P.-M.); wsimpalol@uns.edu.pe (W.D.S.-L.); wcastillo@uns.edu.pe (W.E.C.-M.); lourdes.ep@gmail.com (L.J.E.-P.); 2 Department of Technological Processes and Biotechnology, Institute of Food Science, Technology and Nutrition (ICTAN-CSIC), 28040 Madrid, Spain

**Keywords:** bioactive compounds, bread, digestion, formulation, pseudo-cereals, sprouting

## Abstract

Sprouting is an effective treatment for improving nutritional and bioactive properties as well as lowering the anti-nutritional compounds in pseudo-cereals. Enhancing nutritional properties when using sprouted pseudo-cereals flours as a baking ingredient requires tailored formulation. Simplex centroid designs and response surface methodology has been applied in the present study to define the ideal proportions of ternary blends of sprouted kiwicha (SKF), cañihua (SCF) and wheat flours (WF) to simultaneously enhance the content in bioactive compounds (γ-aminobutyric acid, GABA, total soluble phenolic compounds and TSPC), as well as sensory (odor, color, taste and texture) and functional attributes (antioxidant activity, AA) while reducing phytic acid (PA) content of bread. The effect of gastric and intestinal digestion on bioactive compounds, AA, PA and starch hydrolysis was also evaluated. Mixture design allowed for the identification of optimal formulation (5% SKF, 23.1% SCF, 71.9% WF) that can be used to obtain breads with higher content of GABA, TSPC, AA, overall sensorial acceptability (scores > 7) and reduced PA content and glycemic index. Moreover, this study demonstrated that these nutritional and health benefits provided by the replacement of WF by sprouted pseudo-cereal flours remained upon digestion. The results of this study indicated that WF replacement with SKF and SCF is sensory acceptable and improved the nutritional quality of bread.

## 1. Introduction

It has become increasingly evident that the adoption of a healthy diet is necessary to prevent the risk for disease progression, as well as to maintain environmental sustainability and food security. In this line, industries and consumers have increased the production and consumption for functional foods, which combine low glycemic index (GI), fewer calories and fat content, more plant proteins, fiber and fewer additives and salt according to strongly recommended dietary guidelines [1].

Bread is considered a staple food that is consumed worldwide, with an estimated per capita daily consumption of 180–250 g [2]. In the particular case of bread, fortification with accessible and affordable ingredients with superior sensory, nutritional, and other healthy attributes is an effective strategy aligned with healthy diets and food security [3]. Pseudo-cereals have received attention in this respect thanks to their agronomy traits as well as their remarkable nutritional and technological attributes [4]. Pseudo-cereal grains are edible seeds belonging to dicotyledonous species that are known as such due to their similar physical appearance and high starch content which is similar to true cereals. Two of the most common pseudo-cereals belong to the *Amaranthus* and *Chenopodium* genera. These species are originally from the Andean region and their cultivation could be extended to other parts of the globe as they could grow in harsh climates so that the provision and access to healthy and nutritionally balanced food products containing them could be easily achievable. To this end, fortifying bread formulations with pseudo-cereals could enhance the nutritional value of the final product, specifically increasing the levels of total dietary fiber (TDF), proteins, minerals and bioactive health-promoting compounds while reducing GI and gluten content [3,5]. However, there are still some challenges to overcome in the use of pseudo-cereals in bread making related to the presence of anti-nutrients (saponins with bitter taste, phytic acid [PA]), poor rheological properties of the doughs and sensorial profile of bread [6].

As a sustainable and ancient process, tailored germination (at optimal conditions) could improve not only sensory attributes and reduce anti-nutrients, but also may fully exploit the functional and nutritional attributes of pseudo-cereals. Nowadays, sprouted wholegrain products have appeared as innovative ingredients in the bakery industry. During germination, nutrient seed reserves undergo hydrolysis and mobilization to support seedling growth [7]. Grain germination offers several advantages including a higher nutrient digestibility and bioactive compounds bioavailability [8,9,10]; increased levels of bioactive compounds (e.g., γ-aminobutyric acid [GABA] and phenolic compounds [TSPC]) [8,11,12]; improvement of antioxidant activity (AA) [8,11,12]; and reduced levels of PA [13,14]. The full exploitation of less conventional sprouted grains such as pseudo-cereals in the development of bakery products is limited. Some studies assessed the effect of sprouted pseudo-cereal flours on nutritional, sensory and physicochemical quality parameters of bread [15]. Specifically, 20% sprouted quinoa bread showed a decreased bitterness and, at the same time, the highest specific volume and lowest crumb firmness [16]. Horstman et al. [15] confirmed that flour formulation consisting of sprouted grains (lupin, corn, brown millet, pea, lentil, quinoa and amaranth) produce breads of better quality in comparison to breads from ungerminated grain flours. Moreover, amaranth sprouted flour increased not only the nutritional value but also resulted in the highest loaf volume and softest breadcrumb among the seven types of sprouted grain flours.

Enhancing nutritional properties when using sprouted pseudo-cereals flours as a baking ingredient requires tailored formulation. Simplex centroid designs and response surface methodology has been recently applied to define the ideal proportions of pseudo-cereal (quinoa, buckwheat and amaranth) flours to enhance physical and sensory attributes in gluten-free bread formulations [17]. However, no available reports deal with formulation optimization of a blend of sprouted pseudo-cereal grains from Andean origin such as cañihua (*Chenopodium pallidicaule* Aillen) and kiwicha (*Amaranthus caudatus* L.) in order to improve the content of bioactive compounds and, simultaneously, consumer acceptability and health attributes (e.g., antioxidant activity and GI). Therefore, the objective of this study addressed the improvement of bioactive compounds and sensory and functional attributes of bread through the identification of the most suitable formulation using a simplex-centroid design. Increased GABA, TSPC, AA, odor, taste and texture while reduced PA content in breads were set up as the quality criteria. The effect of gastric and intestinal digestion on bioactive compounds, AA, PA and starch hydrolysis was also evaluated.

## 2. Materials and Methods

### 2.1. Reagents

α-amylase from human saliva, porcine bile extract, pepsin from porcine gastric mucosa, pancreatin from porcine pancreas, 2,2′-diazobis-(2-aminodinopropane)-dihydrochloride (AAPH), fluorescein, fast Blue BB (FBB) [4-(benzoylamino)-2,5-dimethoxybenzenediazonium chloride hemi-(zinc chloride), 6-hydroxy-2,5,7,8-tetramethylchroman-2-carboxylic acid (Trolox) and GABA (>99% purity) were from Sigma-Aldrich, Co. (St. Louis, MO, USA).

### 2.2. Sprouting and Milling Processes

Pseudo-cereal grains cañihua (*Chenopodium pallidicaule* Aillen), kiwicha (*Amaranthus caudatus* L. var. Centenario) originally from the Andean region were supplied by the Cereals and Native Grains Program of Universidad Nacional Agraria La Molina (Peru). Cañihua and kiwicha grains were harvested in 2019 in three different geographical areas. Cañihua was grown in the area of Suni (Puno, Perú) at an altitude of 3827 m. Kiwicha was grown in El Caserío de Huanchacpampa of Carhuaz (Ancash, Perú) at an altitude of 2688 m. Grains were stored at 20 °C in a dry container.

Grains were sprouted at optimal conditions as previously reported to maximize AA, TSPC and GABA [10,12]. Sprouting parameters were summarized in Table S1. Briefly, 300 g of each seed type were soaked in 0.01% sodium hypochlorite for 30 min and then rinsed three to four times with tap water. A second soaking step in sterile water (1:5, *w:v*) was carried out at 23 °C for 7 h. Soaked seeds were placed in trays covered with moist filter paper and incubated in a germination chamber (model BJPX-HT400II, Maquilak, Jinan, China) in the dark with a ≥90% relative humidity.

Sprouted grains were dried in a climatic chamber at 40 °C for 30 h. Dried sprouts were milled in an MDNT-60XL grinding module and passed through a sieve of 0.20 mm pore size (Torrh, Jarcon del Peru S.R.L., Junín, Peru). Two types of flour were obtained [sprouted kiwicha flour (SKF) and sprouted cañihua flour (SCF)] and stored at 4 °C under vacuum in plastic bags.

### 2.3. Bread Making

Experimental bread (BrKC) was prepared using binary combinations of SKF and SCF and wheat flour (WF). A control bread (BrWF) formulated with 100% refined wheat flour (WF) was prepared. WF for bread making (Nicolini, Alicorp S.A. Lima, Peru) with a small particle size (<1 mm) was purchased on the market. Fourteen experimental breads were prepared according to a simplex centroid mixture design, (Table S2). For each formulation 1000 g of flour was added, 2700 g of water, 400 g of seed oil (Primor, Lima, Perú), 100 g of yeast (Universal, Lima, Perú) and 100 g of salt (Lobos, Lima, Perú). For the pre-fermentation, yeast was activated for 10 min. Dough kneading was performed in a k-beater (Nova, Lima, Perú) for 17 min in which disk speed was gradually increased. Afterwards, the kneaded dough was divided into pieces of 650 g that were placed in a proofer for 120 min at 30 °C and 85% relative humidity. After fermentation, doughs were baked at 220 °C for 45 min. Three replicates for each formulation were made. After cooling, the loaves of bread were sealed in polypropylene plastic bags and stored at −20 ± 2 °C.

### 2.4. Simulated Gastrointestinal Digestion

Simulated gastrointestinal digestion of breads was performed following the INFOGEST 2.0 method [18]. Briefly, 3 g of bread were dissolved (1:1 ratio, *w:v*) in simulated salivary fluid (pH 7) containing 75 U/mL of salivary amylase and incubated for 2 min at 37 °C in an orbital shaker (New Brunswick Scientific, Edison, NJ, USA). Oral bolus was mixed (1:1 ratio, *v:v*) with simulated gastric fluid (pH 3) and 2,000 U/mL pepsin solution (final concentration) and incubated for 120 min at 37 °C. Subsequently, gastric digest was diluted with intestinal fluid (1:1 ratio, *v:v*) containing 800 U/mL pancreatin and 10 mM bile and incubated for 120 min. Intestinal digests were thermally treated for enzyme inactivation at 95 °C for 10 min. Digests were freeze-dried (Virtis Company, Gardiner, NY, USA) and stored at −20 °C.

### 2.5. Chemical Characterization of Raw and Sprouted Cañihua, Kiwicha, and Quinoa Flours

Moisture, protein, fat and ash contents were determined using AACC official methods (AACC 44-15A, AACC 46–13, AACC 30–10 and 08–03, respectively) [19]. Starch and phytic acid contents were measured using the enzymatic kits K-TSTA-100A and K-PHYT, respectively, from Megazyme (Wicklow, Ireland). [20]. Samples were analyzed in duplicate and results were expressed in g/100 g of dry weight (dw).

### 2.6. Total Soluble Phenolic Compounds (TSPC)

TSPCs were analyzed according to Pico et al. [21] with slight modifications [22]. Briefly, 50–100 mg of the milled sample containing 1 mL of 80% methanol in 0.1% formic acid were incubated in a Thermomixer at 30 °C and 2000 rpm for 15 min (Eppendorf AG, Hamburg, Germany). After centrifugation in a Centrifuge 5424 R (Eppendorf AG, Hamburg, Germany) for 5 min at 5 °C and 10,000 rpm, the supernatant was collected. A second cycle of extraction with 1 mL of 70% acetone in 0.1% formic acid and centrifugation was performed under the same conditions. Supernatants of the two cycles of extraction were combined and adjusted to a final volume of 2 mL with distilled water. Extract (1 mL) was mixed with 0.1 mL of 0.1% FBBB reagent in distilled water and 0.1 mL of 5% NaOH and incubated for 120 min in the dark at room temperature. After incubation, samples were placed in a microplate and absorbance was read at 420 nm using a Synergy HT microplate reader (BioTek Instruments, Winooski, VT, USA). Gallic acid was used as standard at a concentration range between 2–225 µg/mL. All analyses were performed in duplicate. Data were expressed as mg of gallic acid equivalents (GAE)/100 g of sample dw.

### 2.7. Determination of γ-Aminobutyric Acid (GABA)

Samples were extracted for 30 min by mechanical shaking of 200 mg of sample in 2 mL of 0.1 N HCl using a Thermomixer C (Eppendorf, Madrid, Spain) at 5 °C. Samples were centrifuged for 30 min at 5 °C and 8000× *g* (Centrifuge 5424 R, Eppendorf AG, Hamburg, Germany) and supernatants were filtered using a syringe filter with 0.22 μm nylon membranes. Analysis of GABA in supernatants was performed by reversed-phase high-performance liquid chromatography (RP-HPLC) and UV detection after pre-column derivatization with 9-fluorenylmethoxycarbonyl chloride (FMOC) and *o*-phthaldialdehyde reagents (Agilent, Santa Clara, CA, USA). Chromatographic separations were carried out in an Agilent 1200 high-performance liquid chromatography (Agilent, Santa Clara, CA, USA) equipped with a G1314B diode array detector (DAD), and a Zorbax Eclipse Plus C18 stationary phase column (4.6 × 150 mm, 3 μm). The mobile phase A was composed of 10 mM Na_2_HPO_4_:10 mM Na_2_B_4_O_7_, pH 8.2: 5 mM NaN_3_, and the mobile phase B consisted of acetonitrile:methanol:water (45:45:10, v:v:v). All mobile-phase solvents were HPLC grade. Analyses were performed at 40 °C, with a flow rate of 1.5 mL/min and the following solvent gradient: 57% B in 20 min, 100% B in 20.1 min, 100% B in 23.5 min, 2% B in 23.6 min and 2% B in 25 min. The DAD detector was set to 338 nm (from 0–15 min) and 262 nm (from 15–30 min). External calibration was carried out using standard solutions of GABA (Merck, Madrid, Spain) in the linear range between 10 and 1000 pmol/μL (R^2^ > 0.99). All analyses were performed in duplicate. Results were expressed as mg/100 g dw.

### 2.8. Determination of Oxygen Radical Antioxidant Capacity (ORAC)

The AA was determined by the oxygen radical absorbance capacity (ORAC) method as described previously [15]. Samples extracts were prepared as described in Section 2.6. Briefly, the reaction was performed at 37 °C in 75 mM phosphate buffer at pH 7.4. Aliquots of 30 μL of sample extracts or Trolox (1–160 μM) were placed in a black 96-well plate (Fisher Scientific) and mixed with 180 μL of 70 nM fluorescein, 90 μL of 12 mM 2,2’-azobis(2-amidinopropane) dihydrochloride (AAPH). A kinetic plot of fluorescence decay was plotted by reading fluorescence every minute for 150 min at excitation and emission wavelengths of 485 and 520 nm, respectively, in a Synergy HT microplate reader (BioTek Instruments, Winooski, VT, USA). Results were expressed as μmol Trolox equivalents (TE)/g dw.

### 2.9. Estimation of In Vitro Glycemic Index

*In vitro* starch hydrolysis was determined as described by Goñi et al. [23] with modifications reported by Sanz-Penella et al. [24]. The area under the curve (AUC) was obtained by plotting the starch hydrolysis percentage as a function of digestion time and was used to calculate the hydrolysis index (HI) using white bread as reference (wheat bread; HI = AUC_sample_/AUC_wheat bread_ × 100). The estimated glycemic index (GI) was determined using the equation GI = 0.549 × HI + 39.71.

### 2.10. Acceptability Evaluation

Sensory analysis was performed with thirty participants in three sessions for each experimental design. The panelists assessed the odor, color, taste and texture acceptability of the bread on a semi-structured 10-cm hedonic scale. In each session, bread slices were offered in polyethylene bags randomly coded and delivered in a monadic fashion. Sensory analysis was performed in individual booths in the Sensory Analysis Laboratory of IITA, Universidad Nacional del Santa (Peru). Participants were instructed to drink water between samples to minimize residual effects.

### 2.11. Simplex Centroid Mixture Design

Flour formulation was optimized using the simplex centroid MD for biscuits BQK, BQC and BKQ, respectively. The experimental formulations tested are shown in Table S2. In an experiment with q components, the proportions of the ingredients were denoted by x_1_, x_2_, …, x_q_, where x_i_ ≥ 0 for i = 1, 2, …, q and ∑q_i_ = 1x_i_ = 1, where x_i_ represents the proportion of the i-th component. This equation removes a degree of freedom from the proportions and the factor space is, therefore, a (q−1)-dimensional regular simplex [25]. The design enabled us to approximate the experimental data (Y_obs_) with a response surface model represented in Equations (1)–(4):

Linear ŷ = ∑q_i_ = 1β_i_x_i_, (1)

Quadratic ŷ = ∑q_i_ = 1β_i_x_i_ + ∑_q-1_^i<j^∑q_j_β_ij_x_i_x_j_, (2)

Special cubic ŷ = ∑q_i_ = 1β_i_x_i_ + ∑_q-1_^i<j^∑q_j_β_ij_x_i_x_j_ + ∑_q-2_^i<j^∑_q-1_^j<k^∑q_k_β_ij_kx_i_x_j_x_k_, (3)

Full cubic: ŷ = ∑q_i_ = 1β_i_x_i_ + ∑_q-1_^i<j^∑q_j_β_ij_x_i_x_j_ + ∑_q-1_^i<j^∑q_j_δ_ij_x_i_x_j_(x_i_-x_j_) + ∑_q-2_ i < j∑_q-1_j < k∑q_k_β_ijk_x_i_x_j_x_k_, (4)

The parameter β_i_ represents the expected response to the pure blend x_i_ = 1 and x_j_ = 0 when j ≠ i. The term ∑q_i_ = 1β_i_x_i_ represents the linear blending portion. When curvature arises from non-linear blending between component pairs, the parameters β_ij_, which represent either synergistic or antagonistic blending, will be different from zero [26].

The difference between the experimental data (Y_obs_) and model (Y_calc_) gives the residual (ε). For each response, the R^2^ (squared correlation coefficient) was calculated, which is the fraction of variation of the response explained by the model.

The response variables were PA, GABA, TSPC and ORAC, odor, color, taste and texture. Fourteen formulations were studied with different proportions of cañihua and kiwicha as shown in Table S2.

### 2.12. Statistical Analysis

Three replicates of each experimental formulation were performed while each parameter was analyzed twice per replicate (a data set of 6 values was obtained per sample). Results were expressed as mean ± standard deviation. A T-test (two data sets for comparison of groups treatment vs. control) with posthoc Dunnet’s test or one-way analysis of variance (ANOVA, three or more data sets) with Bonferroni post-hoc test were conducted assuming Gaussian normal distribution and homogeneity of variances. Regression models for the PA, GABA, TSPC, ORAC, odor, color, taste and texture were generated using DoE in the Statistica v.9.0 software (Stasoft, Tulsa, OK, USA). The ANOVA regression models were performed to choose the most significant model (*p* ≤ 0.05) and the best fit (R^2^). Response surfaces and desirability methodology were used to identify optimal formulation.

## 3. Results and Discussion

### 3.1. Comparative Study between Nutritional Composition of Refined Wheat Flours and Sprouted Pseudo-cereal Flours

The chemical composition of WF as compared to SCF and SKF is shown in Table 1. Sprouted pseudo-cereal flours had a better nutritional profile than WF, with significantly lower starch content but higher in TDF, protein, lipids and ashes (*p* ≤ 0.05). Besides being a rich source of dietary fiber as compared to WF, IDF was the main fraction in pseudo-cereals, representing up to 80% of TDF in agreement with other studies [27]. In contrast, soluble dietary fiber (SDF) was the main fraction in WF accounting for 90% of the TDF, with higher values as compared to sprouted pseudo-cereals flours. Although grain germination is known to reduce the content of anti-nutritional compounds [7], sprouted pseudo-cereal flours contained higher amounts of PA with respect to WF. Significant differences in the nutritional composition were observed among sprouted pseudo-cereal flours (*p* ≤ 0.05). SCF showed an outstanding starch, protein, and AA content, as well as lower PA content as compared to SKF (*p* ≤ 0.05). A similar nutritional composition was reported earlier for sprouted cañihua and amaranth flours [5].

Due to the increasing interest in the health-promoting properties of food, GABA, TSPC and AA in raw materials of this study were determined (Table 1). Sprouted pseudo-cereal flours were rich sources of GABA and TSPC and displayed a higher AA as compared to WF (*p* ≤ 0.05). Similar observations were reported by Montemurro et al. [28] in a comparative study of cereal and pseudo-cereal grains and their sprouted counterparts. Significant differences were observed in the content of bioactive compounds and AA between sprouted pseudo-cereal flours, with higher GABA observed for SKF whereas TSPC and ORAC were higher in SCF (*p* ≤ 0.05), in line with earlier studies [9,10,12].

Overall, lower nutritional value of WF as compared to sprouted pseudo-cereal grains could be attributed to the removal of bran outer layers and germ during the refining process of wheat flour [29]. In these anatomical parts of wheat grain, nutrients such as fat, dietary fiber, PA, minerals, vitamins and antioxidant compounds (bound phenolic acids) are concentrated so that they are lost during flour production. On the other side, germination of pseudo-cereal grains is known to increase the digestibility of protein, starch, vitamins, minerals, bioactive compounds (GABA and phenolic compounds) and AA of sprouts [9,10,12]. Particularly, increased GABA amounts in sprouts are attributed to the activation of glutamate-decarboxylase (GAD) during germination, which is involved in the conversion of glutamate to GABA [30]. Similarly, evidence from earlier studies demonstrates that germination of cañihua and kiwicha increase significantly TSPC by the activation of cell wall-degrading enzymes and esterases and up-regulation of biosynthesis routes such as the phenylpropanoid pathway [7]. Additionally, Andean pseudo-cereal grains have consistently shown higher AA in earlier studies as compared to their non-germinated counterparts [9,10,12]. These results were ascribed to the biosynthesis of compounds with AA during germination as a physiological response to maintain a balance of the redox homeostasis and hydrolysis of bound phenolics by cell-wall degrading enzymes [31].

### 3.2. Effect of WF Replacement by Sprouted Pseudo-cereal Flours on Nutritional and Sensory Properties of Breads

Table 2 shows the PA content in BrWF (100% refined WF) and the different BrKC formulations. As compared to BrWF (0.20 g/100 g), the addition of sprouted pseudo-cereal flours in bread formulation increased significantly the amounts of PA reaching between 0.24 and 0.42 g/100 g dw (*p* ≤ 0.05). Bread containing higher amounts of SKF showed higher PA levels in line with the chemical composition of flours shown in Table 1.

The GABA content of the different bread composites varied from 12.4 to 23.6 mg/100 g (Table 2). Significant variations were observed for GABA in some recipes with the same formulation which could be explained by small differences in chemical composition among different germination batches. These values were in the range of cereal-based enriched foods (2.2–16.2 mg/100 g) [32]. The incorporation of sprouted pseudo-cereal flours in the bread formulation improved as much as 2-fold the GABA content as compared to BrWF (11.3 mg/100 g dw). The comparative analysis of bread formulation revealed that the formulations containing higher proportions of SKF stood out in regards to GABA content, which is in line with the results in Table 1.

A significant variation was also observed for the TSPC among different bread formulations. In particular, TSPC varied between 111.9–262.3 mg GAE/100 g (Table 2). Substitution of WF with sprouted pseudo-cereals flour led up to a 5-fold increase of the TSPC as compared to BrWF (46.8 mg GAE/100 g). Higher increases in TSPC were observed in BrKC at the highest level of substitution (11.7% for the total SKF and SCF mixture).

A large variation in the AA of breads was significantly influenced by flour formulation (Table 2). This parameter ranged from 28.8–66.3 μmol TE/g among the different bread formulations, values that were significantly higher than those observed for BrWF (18.5 μmol TE/g). This result suggested that the incorporation of sprouted pseudo-cereal flours in the formulation of breads improved the antioxidant potential of bread in agreement with other studies [33,34]. Among the two types of breads, BrKC, with the highest substitution of SCF, (15%) was characterized to have the highest AA in line with results shown in Table 1.

Sensory properties remain a crucial requirement in food development. For these reasons, all experimental formulations were submitted to a sensory analysis. According to the 10-cm hedonic scale, acceptability scores for odor, color, taste and texture of BrKC formulations varied between 5.41–7.9, 6.9–7.6, 5.84–8.26, 7.11–8.19, respectively. All the studied BrKC formulations were well-accepted for color and texture as non-significant differences were found as compared to BrWF. An opposite result was found for odor and taste in which BrKC formulations with a higher proportion of SKF (recipes #2, 9 and 13) and with 10% SKF and 10% SCF (recipe #13) received lower scores than BrWF (Table 2) indicated they were not well accepted for consumers. For these reasons, sensory and nutritional parameters were all included in the multi-response optimization to identify the optimal formulation with maximum values for all response variables. According to most recent literature, higher proportions of non-sprouted pseudo-cereal flours (60% amaranth, 85% buckwheat and 82% quinoa) in blends with rice flour have been well-accepted in gluten-free breads [17]. When sprouted flours of wheat, barley, chickpea, lentil and quinoa were investigated, all breads received a similar sensory evaluation compared with the non-sprouted counterparts, although raw or sprouted wheat breads were more appreciated [28].

### 3.3. Regression Models and Response Surface Analysis

**Figure 1:**
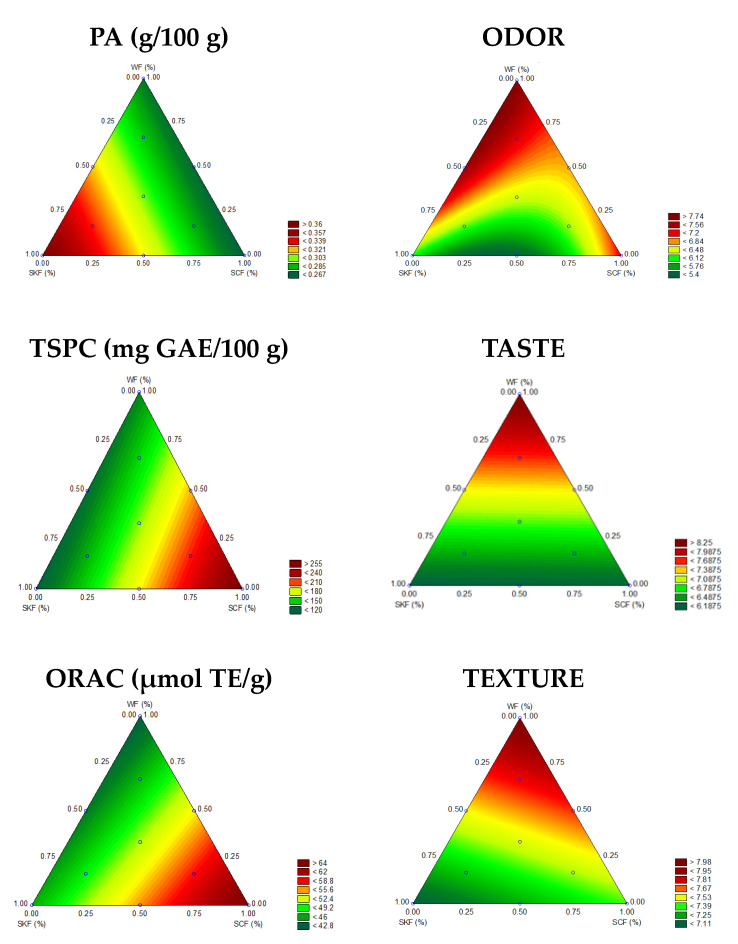
Contour plots of predicted PA, TSPC, ORAC and sensory acceptability scores (10-cm hedonic scale for odor, taste and texture) of breads. Abbreviations: GAE, gallic acid equivalents; ORAC, oxygen radical absorbance capacity; PA, phytic acid; SCF, sprouted cañihua flour; SKF, sprouted kiwicha flour; WF, refined wheat flour; TSPC, total soluble phenolic compounds; TE, Trolox equivalents.

Regression models adjusted to experimental data obtained for nutritional and sensory parameters of experimental breads are presented in Table 3. Only significant models obtained for PA, TSPC and ORAC, odor, taste and texture are included. ANOVA was performed to confirm the confidence of prediction. Linear and quadratic models were obtained, but for some parameters, non-significant binary and ternary interactions were observed for the independent variables included. Significant models at 5% level with a coefficient of determination (R^2^) higher than 0.7 were accepted for predictive purposes [35] and used to generate surface contour plots (Figure 1).

According to linear coefficients, SKF showed the highest coefficient values in the regression model (β_1_: 0.37), therefore, this ingredient is mainly responsible for the hig PA content (Table 3, Figure 1). The variations in TSPC and ORAC in breads have a linear and positive association with flour blends. Higher coefficient values of SCF (β_2:_ 258.03 and 64.73) indicated that this type of flour was responsible for the highest TSPC and ORAC values, respectively (Table 3, Figure 1). Odor, taste and texture variation are mainly explained by the linear effects of bread formulation, although odor variation was also explained by the interaction of binary flour blends (Table 3, Figure 1). The highest acceptability score for odor, taste and texture was associated to higher proportions of WF (β_3_: 7.74, 8.39, 8.00, respectively) and cañihua (Table 3). The contour charts show the feasibility of using different blends of SKF, SCF and WF to obtain BrKC with good acceptability of odor, taste and texture (Figure 1). Blends with higher WF and SCF provide better odor, taste and texture and contribute to higher overall liking.

Desirability function (D) was used to identify optimal bread formulation with the following selected criteria: (1) minimum PA content; (2) maximum GABA, TSPC and AA content; and (3) sensorial acceptability of bread. The optimal formulation with the highest desirability value (D = 0.7) for all studied nutritional and sensorial attributes was 5% SKF, 23% SCF and 72% WF. This optimal formulation was predicted to maintain PA as low as 0.27 g/100 g whereas GABA, TSPC and AA were predicted to reach maximum values of 19.6 mg/100 g and 258.03 mg GAE/100 g and 63.9 μmol TE/g, respectively. In addition, acceptable odor, taste and texture scores were predicted to be nearly or above 7.

### 3.4. Nutritional Properties of Optimised Bread and Effect of In Vitro Digestion

To validate the model, the nutritional characterization of optimized bread was carried out (Table 4) and compared with predicted values (Table 5). The experimental data were in the range of predicted intervals (95% confidence). These results verified the validity of the optimization model. As compared with BrWF, optimized bread contained 6-, 1.6-, 5-, and 3.6-fold higher levels of PA, GABA, TSPC and AA, respectively.

As compared to earlier bread prototypes containing non-sprouted pseudo-cereal grains, the breads developed in this study had lower PA levels than 10% chia, 4% quinoa and 20% amaranth bread (3.4 g/100 g dw) [29].

Regarding GABA content, our results in optimized bread fell in the range reported in the literature for sprouted barley bread (11.9–14.3 mg/100 g) [32,33], sprouted rice bread (37.5 mg/100 g) [34] and sourdough bread (16.8–51.0 mg/100g) [35]. Although in Europe, GABA does not have health claims by EFSA, there is scientific evidence supporting beneficial physiological effects upon intake [36]. GABA is a primary inhibitory neurotransmitter. It has many physiological functions such as a blood pressure regulator, protector of cardiovascular disease and hormonal and cell regulator. In addition, it is associated with brain and psychiatric diseases [32]. GABA could reach the brain in small but sufficient amounts to be of clinical significance [37]. Clinical evidence from 16 studies has demonstrated the antihypertensive effect of GABA (≤10% change in blood pressure) when consumed at doses ranging from 10 mg to 12 mg/day for up to 12 weeks or 120 mg of GABA/day for 12 weeks [38]. Therefore, a serving of 50 g of bread containing pseudo-cereal grains could modestly contribute to reaching the effective daily dose associated with health effects.

Regular dietary intake of polyphenols, approximately 1–2 g per day, has been associated with chronic disease prevention [39]. Although there are no official recommendations for phenolic compounds, it is worth noting that a serving of 50 g of the developed BrKC could cover almost 25% of the reported mean daily intake (1 and 1.2 g/d) [40].

Table 4 shows the effect of in vitro gastric and intestinal digestion of BrWF and optimized bread on PA, GABA, TSPC and AA.

PA content remained unchanged during digestion of BrWF and BrKC (*p* ≥ 0.05). Bio accessible amounts of PA were similar for control and optimized BrKC during gastric and intestinal phases of digestion (*p* ≥ 0.05). In vivo studies have reported PA degradation in the stomach due to activation of endogenous phytases in the food matrix in the acidic environment of the stomach [36]. This effect was not observed in the present study probably due to the thermal inactivation of endogenous phytases during the baking process [37]. Moreover, other studies have reported the degradation of PA during the intestinal digestion of cereal-based foods that was attributed to the phytase activity of applied enzymes and bile extracts used during in vitro digestion [38]. This effect was not observed in our study most probably due to the absence of phytase activity in pancreatin and bile from porcine mucosa.

GABA showed a significant and gradual reduction at the end of gastric and intestinal phases of digestion for BrWF and BrKC, respectively (Table 4). Nonetheless, the amounts of bioaccesible GABA at the end of intestinal digestion were greater for BrKC as compared to BrWF. Resistance of GABA to the gastric environment (pH 1.2) and intestinal conditions was also reported earlier during simulated gastrointestinal digestion of a GABA-rich yogurt in agreement with our results [39,40].

As digestion progressed, the bioaccesible amounts of TSPC markedly increased at the end of gastric and intestinal phase for both BrWF and BrKC. Gastric and intestinal digests of BrKC showed higher amounts of bioaccessible TSPC as compared to BrWF. Consistent with TSPC, AA increased at the end of gastric and intestinal endpoints in both breads, with higher values observed for BrKC digests than BrWF, regardless of time endpoint. Similar results were reported by Hidalgo et al. [41] who observed an increased TSPC and AA in gastric and intestinal digests of different bread formulations (ternary blends of wheat and einkorn mixture with quinoa, buckwheat and amaranth) as compared to non-digested samples. At the end of digestion, Hidalgo et al. [41] reported similar TSPC and AA content for all the bread digests studied. Consistently, Bacek et al. [42] noticed increased amounts of TSPC and AA after the gastrointestinal digestion of breads with oat/buckwheat binary blends or 100% flour. However, in this study by Bacek et al. [42]*,* breads formulated with 100% buckwheat showed the highest levels of bioaccessible phenolic compounds and AA at the end of digestion.

The starch content in optimized bread was significantly lower than BrWF (Table 4). Moreover, the starch digestion kinetics and GI of optimized BrKC were compared to BrWF (Table 4, Figure S1). The in vitro starch hydrolysis was evaluated by plotting the % of starch hydrolyzed as a function of digestion time (Figure S1). The highest hydrolysis rate was between 0 and 5 min and with lower rates thereafter for both breads (Figure S1). The hydrolysis plateaus were reached at 40 min and lower HI (79.14 ± 1.06) was observed for BrKC (5% SKF, 23.1% SCF, 71.9% WF) than for BrWF (96.7 ± 1.54%) (Table 4). Consequently, predicted GI significantly decreased in BrKC (83.16) compared to BrWF (92.81). This variability reflects different rates of starch digestion, where the porous structure of BrWF is easily destructed in the oral, gastric and intestinal phases, leading to a high glycemic response [43] as seen by very high hydrolysis rates (Figure S1). Our results are aligned with the existing literature. For instance, gluten-free bread prepared with 100% buckwheat, oat, quinoa, sorghum, and teff flours all had lower GI (between 72–85) than that of the reference white wheat bread [44]. The reason for the difference between BrWF and BrKC could rely on differences in chemical composition. Protein and fiber are macromolecules known to mitigate GIs in food preparations in bread [45]. SKF and SCF contained a higher content of protein and fiber than WF (Table 1) which could explain the lower GIs of BrKC compared to BrWF. The presence of TDF in pseudo-cereals can influence the glycemic response due to its physicochemical properties in the gastrointestinal tract reducing both glucose and insulin responses [43]. Moreover, enrichment of bread in phenolic compounds could result in the inhibition of α-amylase activity [44].

## 4. Conclusions

This work disclosed that it is feasible to produce breads with improved nutritional and health benefits through a fortification strategy based on the replacement of WF with SKF and SCF. The mixture design allowed for the identification of optimal formulation (5% SKF, 23.1% SCF, 71.9% WF) that can be used to obtain breads with higher content of GABA, TSPC, AA, overall sensorial acceptability (scores > 7) and lower PA content and GI. Moreover, this study demonstrated that these nutritional and health benefits provided by the replacement of WF by sprouted pseudo-cereal flours remained upon digestion. Considering the popularity of bread around the world, this information should be taken into account to transform bread into a more nutritive and healthier product so as to positively influence public health.

## Figures and Tables

**Table 1:** Chemical composition (expressed as dry weight, dw) of wheat flour and sprouted pseudo-cereal flours.

Parameters	WF	SCF	SKF
Starch (g/100 g dw)	71.63 ± 0.77 ^c^	41.21 ± 1.47 ^b^	32.45 ± 0.85 ^a^
TDF (g/100 g dw)	8.50 ± 0.78 ^a^	22.06 ± 0.83 ^b^	23.06 ± 0.67 ^b^
IDF (g/100 g dw)	0.73 ± 0.26 ^a^	15.28 ± 0.95 ^b^	16.18 ± 0.60 ^b^
SDF (g/100 g dw)	7.77 ± 0.53 ^b^	6.88 ± 0.12 ^a^	6.87 ± 0.07 ^a^
Protein (g/100 g dw)	12.63 ± 0.03 ^a^	19.11 ± 0.27 ^c^	15.38 ± 0.11 ^b^
Fat (g/100 g dw)	1.02 ± 0.10 ^a^	6.23 ± 0.25 ^b^	5.86 ± 0.38 ^b^
Ash (g/100 g dw)	0.56 ± 0.01 ^a^	2.68 ± 0.05 ^b^	2.85 ± 0.08 ^b^
PA (g/100 g dw)	0.19 ± 0.00 ^a^	0.88 ± 0.01 ^b^	1.24 ± 0.02 ^c^
GABA (mg/100 g dw)	13.67 ± 0.48 ^a^	100.00 ± 22.45 ^b^	217.98 ± 1.48 ^c^
TSPC (mg GAE/100 g dw)	63.97 ± 4.53 ^a^	386.12 ±27.83 ^c^	144.72 ± 2.09 ^b^
ORAC (μmol TE/g dw)	18.55 ± 4.02 ^a^	114.92 ± 14.17 ^c^	35.44 ± 4.55 ^b^

Data are means ± standard deviation of three replicates. Different letters denote statistical differences among samples (ANOVA, Bonferroni *post-hoc* test, *p* ≤ 0.05). Abbreviations: dw, dry weight; GABA, γ-aminobutyric acid; GAE, gallic acid equivalents; IDF: insoluble dietary fiber; ND: not detected; ORAC, oxygen radical absorbance capacity; PA, phytic acid; SCF, sprouted cañihua flour; SKF, sprouted kiwicha flour; SDF, soluble dietary fiber; TSPC, total soluble phenolic compounds; TDF, Total dietary fiber; TE, Trolox equivalents; WF, refined wheat flour.

**Table 2:** Effect of flour formulation with ternary blends of WF, SKF and SCF on nutritional and sensory parameters of bread.

Bread Type	Recipe no.	Proportion of Flours ^a^			PA (g/100 g)	GABA (mg/100 g)	TSPC (mg GAE/100 g)	ORAC (μmol TE/g)	ODOR	COLOR	TASTE	TEXTURE
		SKF	SCF	WF								
BrKC	1	8.33	8.33	83.33	0.26 ± 0.03 ^b^	16.45 ± 0.25 ^bc^	173.34 ± 12.59 ^de^	56.55 ± 1.47 ^de^	6.68 ± 1.46 ^bcde^	7.41 ± 1.20 ^a^	6.32 ± 1.32 ^abc^	7.32 ± 1.49 ^a^
	2	15	5	80	0.30 ± 0.03 ^bcd^	19.08 ± 0.88 ^def^	123.24 ± 11.65 ^bc^	47.62 ± 3.81 ^cd^	6.04 ± 1.15 ^ab^	7.13 ± 1.46 ^a^	6.48 ± 1.31 ^abc^	7.28 ± 1.25 ^a^
	3	10	10	80	0.30 ± 0.05 ^bcd^	19.39 ± 0.09 ^def^	159.89 ± 2.77 ^cde^	60.40 ± 6.67 ^de^	5.41 ± 1.30 ^a^	6.95 ± 1.44 ^a^	5.84 ± 1.57 ^a^	7.11 ± 1.43 ^a^
	4	5	15	80	0.25 ± 0.01 ^b^	18.21 ± 0.06 ^cde^	237.46 ± 1.58 ^fg^	66.30 ± 4.51 ^e^	7.01 ± 1.50 ^bcdefg^	7.48 ± 1.19 ^a^	6.28 ± 1.41 ^abc^	7.41 ± 1.26 ^a^
	5	5	15	80	0.30 ± 0.01 ^bcd^	18.62 ± 0.05 ^de^	251.17 ± 3.36 ^g^	58.09 ± 2.23 ^de^	7.26 ± 1.38 ^cdefg^	7.56 ± 1.36 ^a^	6.14 ± 1.35 ^ab^	7.69 ± 1.20 ^a^
	6	5	10	85	0.25 ± 0.02 ^b^	16.52 ± 0.53 ^bc^	200.46 ± 1.20 ^ef^	51.40 ± 4.47 ^cd^	6.62 ± 1.29 ^bcde^	7.13 ± 1.37 ^a^	7.40 ± 1.30 ^cdef^	7.92 ± 1.07 ^a^
	7	6.67	11.67	81.67	0.27 ± 0.01 ^bc^	19.69 ± 0.30 ^ef^	262.30 ± 15.89 ^g^	53.43 ± 0.86 ^de^	6.42 ± 1.09 ^abcd^	7.08 ± 1.23 ^a^	6.82 ± 1.13 ^abcd^	7.15 ± 1.23 ^a^
	8	5	5	90	0.24 ± 0.01 ^b^	12.39 ± 0.67 ^a^	112.15 ± 12.49 ^b^	38.79 ± 1.19 ^bc^	7.87 ± 1.38 ^fg^	7.39 ± 1.14 ^a^	8.53 ± 1.11 ^f^	7.90 ± 1.30 ^a^
	9	15	5	80	0.42 ± 0.01 ^e^	17.63 ± 0.53 ^cd^	111.96 ± 7.76 ^b^	39.37 ± 0.59 ^bc^	6.31 ± 1.43 ^abc^	7.24 ± 1.20 ^a^	6.32 ± 1.20 ^abc^	7.22 ± 1.29 ^a^
	10	10	5	85	0.35 ± 0.00 ^cde^	15.70 ± 0.19 ^b^	116.63 ± 0.59 ^b^	49.09 ± 0.04 ^cd^	7.56 ± 1.31 ^defg^	7.14 ± 1.19 ^a^	7.16 ± 1.18 ^bcde^	7.45 ± 1.31 ^a^
	11	6.67	6.67	86.67	0.28 ± 0.01 ^bc^	19.48 ± 0.18 ^def^	165.17 ± 0.31 ^cde^	51.64 ± 0.53 ^cd^	7.27 ± 1.20 ^cdefg^	6.91 ± 1.11 ^a^	7.67 ± 1.22 ^def^	7.81 ± 1.53 ^a^
	12	5	10	85	0.31 ± 0.01 ^bcd^	20.51 ± 0.08 ^f^	196.78 ± 18.70 ^ef^	55.79 ± 0.97 ^de^	6.73 ± 1.30 ^bcdef^	6.90 ± 1.18 ^a^	7.63 ± 1.16 ^def^	7.57 ± 1.13 ^a^
	13	11.67	6.67	81.67	0.36 ± 0.01 ^de^	23.62 ± 0.50 ^g^	149.75 ± 13.42 ^bcd^	48.74 ± 3.29 ^cd^	5.90 ± 1.18 ^ab^	7.05 ± 1.21 ^a^	6.40 ± 1.13 ^abc^	7.21 ± 1.08 ^a^
	14	5	5	90	0.30 ± 0.01 ^bcd^	15.48 ± 0.42 ^b^	131.18 ± 8.20 ^bcd^	46.86 ± 2.94 ^cd^	7.61 ± 1.29 ^efg^	7.19 ± 1.17 ^a^	8.26 ± 1.36 ^ef^	8.19 ± 1.18 ^a^
BrWF	15	0	0	100	0.20 ± 0.01 ^a^	11.26 ± 0.40 ^a^	46.80 ± 3.47 ^a^	18.45 ± 1.82 ^a^	7.95 ± 1.04 ^g^	7.89 ± 0.92 ^a^	7.91 ± 0.97 ^def^	8.03 ± 0.85 ^a^

Data are means ± standard deviation of three replicates. Different letters show statistical differences among different formulations (ANOVA, Bonferroni *post hoc* test, *p* ≤ 0.05). Abbreviations: BrKC, breads formulated with sprouted kiwicha (SKF), sprouted cañihua (SCF) and wheat flour (WF); BrWF, bread formulated with 100% refined wheat flour; GABA, γ-aminobutyric acid; GAE, gallic acid equivalents; ORAC, oxygen radical absorbance capacity; PA, phytic acid; TSPC, total soluble phenolic compounds; TE, Trolox equivalents.

**Table 3:** Predictive regression models describing the relationships between the nutritional attributes of the breads and flour blend composition.

Dependent Variables	Mathematical Models	R^2^ (pred)	R^2^ (adj)
PA	ŷ = + 0.36X_1_ + 0.27X_2_ + 0.27X_3_	0.80	0.77
TSPC	ŷ = +117.02X_1_ + 258.03X_2_ + 131.84X_3_	0.87	0.85
ORAC	ŷ = +46.40X_1_ + 64.73X_2_ + 42.54X_3_	0.75	0.70
ODOR	ŷ = 6.12X_1_ + 7.15 X_2_ + 7.74X_3_ − 4.81X_1_ X_2_ + 2.52 X_1_ X_3_ − 2.79X_2_ X_3_	0.95	0.92
TASTE	ŷ = 6.16 X_1_ + 6.21 X_2_ + 8.39X_3_	0.90	0.89
TEXTURE	ŷ = 7.11 X_1_ + 7.40X_2_ + 8.00X_3_	0.72	0.70

Abbreviations: BrKC bread formulated with sprouted kiwicha (X_1_), sprouted cañihua (X_2_) and wheat flour (X_3_); ORAC, oxygen radical absorbance capacity; PA, phytic acid; TSPC, total soluble phenolic compounds.

**Table 4:** In vitro starch digestibility and changes in PA, GABA, TSPC and AA at different phases of digestion for BrWF (100% WF) and BrKC with optimal formulation (5% SKF, 23.1% SCF, and 71.9% WF).

Digestion Phase/Time	Parameters	BrWF	BrKC
None/0 min	PA (g/100 g)	0.20 ± 0.01 ^a^	0.25 ± 0.01 ^a,^*
	GABA (mg/100 g)	11.26 ± 0.40 ^a^	18.21 ± 0.60 ^a,^*
	TSPC (mg GAE/100 g)	46.80 ± 3.47 ^a^	237.46 ± 1.58 ^a,^*
	ORAC (μmol TE/g)	18.45 ± 1.82 ^a^	66.06± 3.21 ^a,^*
Gastric/120 min	PA (g/100 g)	0.26 ± 0.02 ^a^	0.23 ± 0.02 ^a^
	GABA (mg/100 g)	9.67 ± 0.90 ^a^	17.00 ± 0.24 ^a,^*
	TSPC (mg GAE/100 g)	202.44 ± 16.13 ^b^	362.96 ± 4.47 ^b,^*
	ORAC (μmol TE/g)	38.40 ± 1.84 ^b^	93.74 ± 6.17 ^b,^*
Intestinal/120 min	PA (g/100 g)	0.26 ± 0.01 ^a^	0.26 ± 0.03 ^a^
	GABA (mg/100 g)	11.7 ± 1.28 ^a^	18.5 ± 1.02 ^a,^*
	TSPC (mg GAE/100 g)	263.11 ± 4.21 ^c^	357.18 ± 4.38 ^b^
	ORAC (μmol TE/g)	45.33 ± 5.42 ^c^	109.11 ± 8.71 ^c,^*
In vitro starch digestibility	Starch (g /100 g)	55.89 ± 0.27	45.52 ± 0.96 *
	HI	96.7 ± 1.54	79.14 ± 1.06 *
	AUC	31,642.5 ± 999.3	25,042.2 ± 336.7 *
	GI	92.81 ± 0.85	83.16 ± 0.58 *

Data are means ± standard deviation of three replicates. * Denote statistical differences between mean values of control and BrKC data sets (Dunnet’s test, *p* ≤ 0.05). Different letters show statistical differences among mean values at different phases of digestion (ANOVA, Bonferroni post-hoc test, *p* ≤ 0.05). Abbreviations: AUC, area under the curve; BrKC, bread formulated with sprouted kiwicha, sprouted cañihua and wheat flours; GABA, γ-aminobutyric acid; GAE, gallic acid equivalents; GI, glycemic index; HI, hydrolysis index; ORAC, oxygen radical absorbance capacity; PA, phytic acid; SCF, sprouted cañihua flour; SKF, sprouted kiwicha flour; SQF, sprouted quinoa flour; TSPC, total soluble phenolic compounds; TE, Trolox equivalents; WF, refined wheat flour.

**Table 5:** Composition of flour blends and predicted values for PA, GABA, TSPC and ORAC in breads at optimum desirability value (D).

Optimum Desirability Value (D)	Optimal Formulation	Response Variables	Predicted Values	−95% CI	+95% CI
0.704	5% SKF, 23% SCF, 72% WF	PA (g/100 g)	0.27	0.24	0.32
		GABA (mg/100 g)	19.58	12.39	23.62
		TSPC (mg GAE/100 g)	258.03	211.96	262.30
		ORAC (μmol TE/g)	63.88	58.78	66.30
		Odor	7.10	6.41	7.37
		Taste	7.2	6.84	7.53
		Texture	7.62	7.51	8.09

Abbreviations: CI, confidence interval; GABA, γ-aminobutyric acid; GAE, gallic acid equivalents; ORAC, oxygen radical absorbance capacity; PA, phytic acid; SCF, sprouted cañihua flour; SKF, sprouted kiwicha flour; TSPC, total soluble phenolic compounds; TE, Trolox equivalents; WF, refined wheat flour.

## Data Availability

The data presented in this study are available in this article or Supplementary Materials.

## Supplementary Materials

The following supporting information can be downloaded at: https://www.mdpi.com/article/10.3390/foods11111541/s1, Figure S1: Starch hydrolysis kinetic of BrWF (100% WF) and BrKC (5% SKF, 23% SCF, 72% WF). Table S1: Sprouting parameters of cañihua and kiwicha grains. Table S2: Experimental design with three independent variables (proportion of flour blends).
