# Supplementary material for: Reformulating Bread Using Sprouted Pseudo-cereal Grains to Enhance Its Nutritional Value and Sensorial Attributes
Source: Foods. 2022 May 24;11(11):1541. doi: 10.3390/foods11111541 (PMC9180012; doi:10.3390/foods11111541)
Supplement: Supplementary file 1 [file foods-11-01541-s001.zip › foods-1743090-supplementary.pdf]

Table S1. Sprouting parameters of cañihua and kiwicha grains.

Grains	Temperature (°C)	Time (h)	Germination rate (%)
Cañihua	20	72	98
Kiwicha	26	63	100

The optimal germination temperature and time of cañihua and kiwicha was established based on previous optimization studies [1,2].

Table S2. Experimental design with three independent variables (proportion of flours blends)

Recipe no.	Proportion of flours[a]		
	Sprouted kiwicha flour (X_1)	Sprouted cañihua flour (X_2)	Wheat flour (X_3)
1	8.33	8.33	83.33
2	15	5	80
3	10	10	80
4	5	15	80
5	5	15	80
6	5	10	85
7	6.67	11.67	81.67
8	5	5	90
9	15	5	80
10	10	5	85
11	6.67	6.67	86.67
12	5	10	85
13	11.67	6.67	81.67
14	5	5	90

[a] Weight proportions equivalent to the original flour (expressed as %; sum of ingredients = 100%) used in bread preparations.

Figure S1. Starch hydrolysis kinetic of BrWF (100% WF) and BrKC (5% SKF, 23% SCF, and 72% WF)

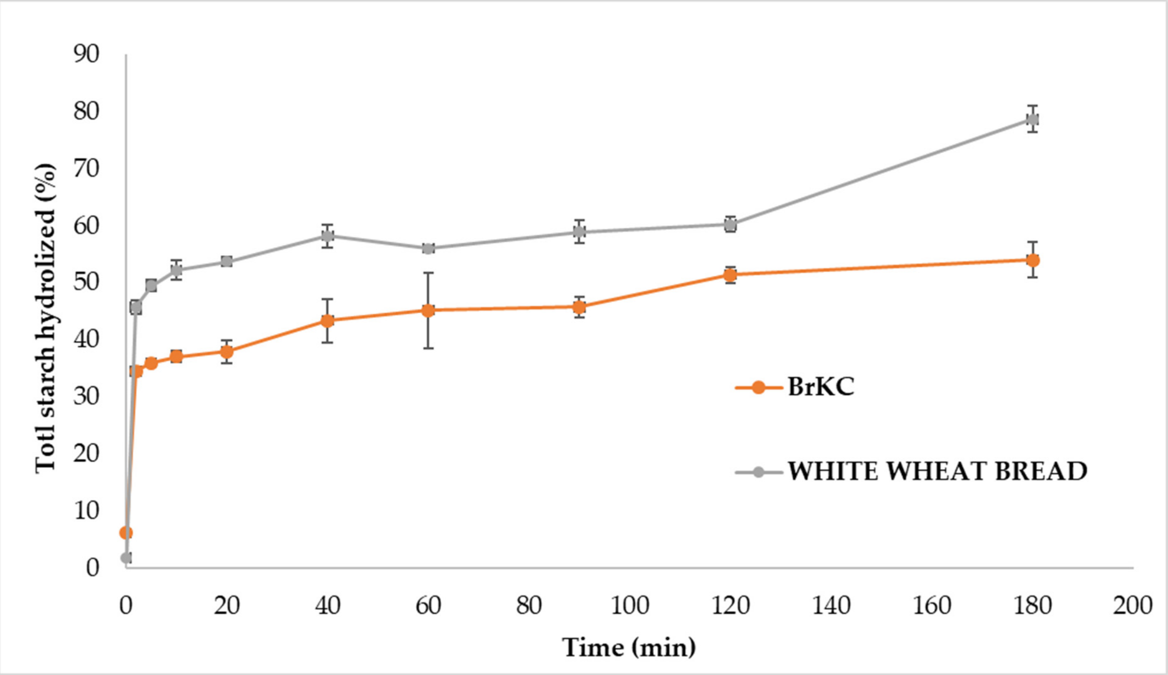

Abreviations: BrKC, bread formulated with 5% sprouted kiwicha, 23% sprouted cañihua and 72% wheat flours; BrWF: bread formulated with 100% refined wheat flour; SCF: sprouted cañihua flour; SKF, sprouted kiwicha flour; WF: refined wheat flour.

References

1. Paucar-Menacho, L.M.; Peñas, E.; Dueñas, M.; Frias, J.; Martínez-Villaluenga, C. Optimizing germination conditions to enhance the accumulation of bioactive compounds and the antioxidant activity of kiwicha (Amaranthus caudatus) using response surface methodology. *LWT - Food Science and Technology* **2017**, *76*, 245-252, doi:10.1016/j.lwt.2016.07.038.
2. Abderrahim, F.; Huanatico, E.; Repo-Carrasco-Valencia, R.; Arribas, S.M.; Gonzalez, M.C.; Condezo-Hoyos, L. Effect of germination on total phenolic compounds, total antioxidant capacity, Maillard reaction products and oxidative stress markers in canihua (Chenopodium pallidicaule). *Journal of Cereal Science* **2012**, *56*, 410-417, doi:https://doi.org/10.1016/j.jcs.2012.04.013.
